# Widespread tsunami-like waves of 23-27 June in the Mediterranean and Black Seas generated by high-altitude atmospheric forcing

**DOI:** 10.1038/srep11682

**Published:** 2015-06-29

**Authors:** Jadranka Šepić, Ivica Vilibić, Alexander B. Rabinovich, Sebastian Monserrat

**Affiliations:** 1 Institute of Oceanography and Fisheries, Šetalište I. Meštrovića 63, 21000 Split, Croatia; 2 P.P. Shirshov Institute of Oceanology, Russian Academy of Sciences, 36 Nakhimovsky Pr., Moscow, Russia; 3 Fisheries and Oceans Canada, Institute of Ocean Sciences, 9860 W. Saanich Rd., Sidney, BC, Canada; 4 Department of Physics, University of the Balearic Islands, Ctra.Valldemossa, km. 7.5, Palma de Mallorca, Spain

## Abstract

A series of tsunami-like waves of non-seismic origin struck several southern European countries during the period of 23 to 27 June 2014. The event caused considerable damage from Spain to Ukraine. Here, we show that these waves were long-period ocean oscillations known as meteorological tsunamis which are generated by intense small-scale air pressure disturbances. An unique atmospheric synoptic pattern was tracked propagating eastward over the Mediterranean and the Black seas in synchrony with onset times of observed tsunami waves. This pattern favoured generation and propagation of atmospheric gravity waves that induced pronounced tsunami-like waves through the Proudman resonance mechanism. This is the first documented case of a chain of destructive meteorological tsunamis occurring over a distance of thousands of kilometres. Our findings further demonstrate that these events represent potentially dangerous regional phenomena and should be included in tsunami warning systems.

A *chain of destructive tsunami-like events* took place during the period 23–27 June 2014 in the Mediterranean and the Black Sea regions, affecting countries from Spain to Ukraine (Fig. 1). The sudden occurrence, height, destructiveness, and run-up of the observed waves all indicate that these were tsunami-like events. Great earthquakes or volcanic eruptions can lead to the generation of tsunami waves of such great spatial extent. However, even when crossing vast oceanic regions, these waves do not take longer than 48 hours to arrive at the most distant locations^1^,^2^. Landslides or atmospheric disturbances can also induce tsunamis but they typically affect limited regions^3^,^4^. In this study, we show that tsunami waves observed along the coasts of a number of southern European countries in the last week of June 2014 had an atmospheric origin, and were therefore a series of individual meteorological tsunamis (“meteotsunamis”)^4^. The extraordinary expanse of this event shows that meteotsunamis can have a widespread influence that is spatially comparable to other major tsunamigenic mechanisms.

A few hours after midnight on 22/23 June, 1-meter tsunami-like oscillations were observed in Ciutadella Inlet on coast of the Balearic Islands (Spain). On 25 and 26 June, several tsunami-like waves with heights of up to 3 m struck a number of bays and harbours in the central and south Adriatic Sea. The strongest oscillations occurred in the morning of 25 June at the head of the 8-km long Vela Luka Bay. Here at approximately 6:35 UTC, sea level rapidly reached +1.5 m and then, 10 min later, fell to −1.5 m relative to ambient sea level. (It was in this bay that a catastrophic meteotsunami had induced serious flooding in June 1978^5^). Later the same day (at 11:00–15:00 UTC), oscillations similar to those in Vela Luka Bay with wave heights of up to 2.5 m and strong (~10 knots) currents were observed in other bays within the Adriatic located ~30–120 km from Vela Luka^6^. Intense tsunami-like waves also impacted the southwestern coast of Sicily at approximately 19:00 UTC on 25 June. The highest waves were observed at the mouth of the Mazara River, where a powerful wave created a destructive 1.5 m high hydraulic jump (bore) that significantly damaged a number of boats moored in the harbour^7^. Large seiche oscillations were also observed on 25–26 June at other coastal regions of the Central and Eastern Mediterranean, including the coasts of Italy, Greece and Turkey. Finally, at noon on 27 June 2014 during a calm summer day, a 1 to 2 m high tsunami-like wave struck the beaches of Odessa and the neighbouring port town of Illichevsk in the northwestern Black Sea (Ukraine). Six people, including four children, were injured and had to be transported to a local hospital.

All known tsunamis observed in the Mediterranean and Black seas were generated by earthquakes with magnitudes *M*_w_ > 5.5^8^,^9^, while at the time of June 2014 events, the entire region was seismically quiet (*M*_w_ < 2.7). Moreover, these regions have not typically been associated with landslide-generated tsunamis. We, therefore, conclude that all the events that occurred during the June 2014 time period were *meteorological tsunamis* (“meteotsunamis”)^4^, long destructive tsunami-like waves generated by atmospheric disturbances (atmospheric gravity waves, pressure jumps, frontal passages, squalls)^4^,^10^. This phenomenon is known in the Mediterranean Sea region as “*rissaga*” (Balearic Islands)^4^, “*šćiga*” (Adriatic Sea)^5^, and “*marrubbio*” (Sicily)^4^,^11^. Major meteotsunamis in this region are usually not associated with extreme atmospheric events such as hurricanes or major storms, but with marginally detectable changes in atmospheric pressure often caused by atmospheric gravity waves that are frequently present during times of calm weather, as was the case at the four locations. Meteotsunami formation is related to very specific and comparatively rare *resonant situations* that lead to strong amplification of the initial open-seawaves^4^,^10^. Ciutadella Inlet on Menorca Island (Spain)^12^, Vela Luka Bay on Korčula Island (Croatia)^5^,^13^, and Mazara del Vallo harbour on the western coast of Sicily^11^ are locations in the Mediterranean where meteotsunamis (resembling extreme *seiches*) occur most often and have anomalously large heights (up to 3–6 m). Meteotsunamis are less common in the Black Sea, but there were several historical events on the coast of Turkey described as “tsunamis of unknown origin”^9^, which could have been meteotsunamis. Also, an extraordinary event identified as meteotsunami occurred in May 2007 in western Black Sea, when 2–3 m high tsunami waves hit the northern Bulgarian coastline. The event was associated with sudden air pressure changes that occurred during relatively calm surface weather^14^. In most other countries on the Black Sea coast, including Ukraine and Russia, meteotsunamis are almost unknown. For this reason, the Odessa event of 27 June 2014 gave rise to considerable concern and misinformation, due primarily to a lack of a prompt scientific explanation. Rumours about the possible causes for the waves spread among the general public and a number of unfounded explanations, including an underwater explosion, ship (submarine) waves, whirlwind effect, extreme interacting currents, abrupt temperature changes and even ”the great cross of planets” surfaced in the media. This differed from locations in the Mediterranean where most media reports stated succinctly that “meteotsunamis were hitting once again!”.

Figure 2 shows simultaneous pairs of 3-h cut-off, high-pass filtered time series of air pressure and sea level recorded at coastal locations on the Balearic Islands (Spain), in the Adriatic Sea (Croatia), and in Sicily (Italy). Corresponding frequency-time (*f-t*) diagrams are also shown for each of these series. All sets of figures show that the passage of pronounced atmospheric disturbances is roughly concurrent with the onset of strong sea level oscillations. Although the linkages between enhanced sea level oscillations and intensified atmospheric disturbances are highly non-linear, the time series are clearly indicative of a cause and effect relationship. These relationships become more apparent if we compute the correlation between time series of air pressure and sea level variance. Variance time series are representative of temporal changes in the signal kinetic energy. For the co-located air pressure and sea level stations on the Spanish coast, there is a high maximum correlation coefficient, *r* = 0.91, between the variance series at zero time lag. In contrast, the atmospheric pressure data for Croatia and Italy are from locations relatively far removed from their corresponding coastal sea level stations (~100 km separation between sites), so that the correlation coefficients at zero lag are considerably lower than for the Menorca site. To demonstrate a cause-and-effect linkage for the Croatian and Italian sites, the atmospheric wave direction and speed must be taken into account. If we adjust the timing of the Croatian air pressure record by ~2 hours, we obtain a maximum *r* = 0.65; a similar adjustment of the Italian record by 19 min leads to a maximum *r* = 0.56. The adjusted results for all three sites are consistent with sea level oscillations generated by passing atmospheric events. There are no similar high-quality data for the area of Odessa but air pressure records from Constanta and Mangalia (Romania), located 300–350 km southwest of Odessa, clearly show a train of atmospheric disturbances, similar to those in the Mediterranean, passing by these stations at 04:00–06:00 UTC on 27 June. It is possible that these, or related disturbances, were responsible for the “Odessa tsunami” observed at approximately 09:50 UTC. The air pressure records and a segment of an analogue tide gauge record at Odessa are shown in the inset of Fig. 1.

Thus, available data indicate a meteorological origin for the observed series of tsunami-like waves in the Mediterranean and Black Sea regions on 23–27 June 2014. All previously determined hazardous meteotsunamis, except those associated with hurricanes or similar large-scale atmospheric structures, were *local events* that were observed in one or two neighbouring bays or at a particular confined beach^4^,^15^. The June 2014 event represents the first known case of a succession of individual meteotsunamis sequentially affecting several countries located hundreds to thousands of kilometres apart. The eastward progression of meteotsunami occurrence (Fig. 1) points to a possible link with weather systems, which predominantly propagate eastward over the Mediterranean. At the same time, it is well established that meteotsunamis are usually generated by short-term, small-scale (horizontal dimensions of ~100 km) disturbances^4^,^16^ that normally exist only for a few hours and cannot propagate over long distances. Comparison of air pressure records from various sites during the 2014 event (Fig. 2), reveals that the atmospheric disturbances were similar but not identical.

It appears that the extended June 2014 event was the result of anomalous atmospheric conditions over the Mediterranean/Black Sea region. These conditions supported the generation of numerous intense, small-scale atmospheric disturbances (we refer to this state as a “tumultuous atmosphere”), which subsequently triggered the meteotsunamis. Numerous studies in the vicinity of the Balearic Islands and the Adriatic Sea demonstrate that meteorological tsunamis typically occur during warm seasons when the following conditions are satisfied^12^,^13^,^17^: (1) inflow of warm and dry air from Africa at heights of ~850 hPa (~1500 m); (2) a strong south-westerly jet stream (with wind speeds >20 m/s) at heights of ~500 hPa (~5000 m); and (3) the presence of unstable atmospheric layers (at heights of 600–400 hPa) characterized by a small Richardson number, *Ri* <0.25. An atmospheric pattern favourable to meteotsunami generation was tracked propagating eastward from 23 to 27 June (Fig. 3). The pattern was first observed over the Balearic Islands at the time of the Ciutadella event (23 June, 00:00 UTC). The system then propagated to the east, reaching its full strength over the Adriatic and Tyrrhenian seas and the Strait of Sicily. Jet-stream speeds at the time were greater than 40 m/s and were accompanied by broad unstable atmospheric areas. Major meteotsunami events were observed exactly at the time of the most intense atmospheric instability and a well-developed jet-stream over each respective area (Fig. 3). As it moved further to the east, the system weakened. Before it completely faded away, however, it arrived at the northwestern Black Sea region coincident with the time of the Odessa event (27 June, at ~12:00 UTC). Amplification and attenuation of high-frequency sea level oscillations consistent with the travel times of the synoptic pattern can also be tracked from a number of the Mediterranean tide-gauge records (Fig. 3).

It seems likely that the moving synoptic pattern was responsible for the continual generation of small-scale atmospheric pressure perturbations that would have been forming and then collapsing as they drifted with the jet-stream. In turn, these numerous atmospheric disturbances generated ubiquitous tsunami-like waves, which became destructive in specific areas. Strong horizontal gradients in the jet-stream, like the gradients observed during 23–27 June, are known to be places where atmospheric disturbances (in particular atmospheric gravity waves) are generated^18^. Normally, atmospheric gravity waves dissipate before travelling one full wavelength^19^, and therefore do not have sufficient time to produce significant sea level oscillations. However, under particular atmospheric conditions, the induced internal gravity waves are trapped, leading to the formation of so-called “ducted waves” which maintain their shape and intensity as they propagate over relatively long distances^19^,^20^. A schematic presentation of a ducted wave along with meteotsunami generation mechanism is provided in Fig. 4. Provided there is an overlying unstable layer (*Ri* < 0.25) that contains a critical (steering) level in which the wind speed, *U*, equals the propagation speed of ducted waves^19^,^20^, ducted waves with speed *U* can become trapped in a stable atmospheric layer adjacent to the ground. If there were no unstable layer, wave energy would radiate vertically, and if there were no steering layer, wave energy would be absorbed rather than reflected^19^. During the above synoptic conditions, both the generation and subsequent trapping of atmospheric gravity waves are supported: Dry African air increases the stability of the lower atmospheric layer (elevations of up to 4000–5000 m), whereas strong vertical wind shear and moist advection from the Atlantic Ocean generate an unstable layer which serves as the generating, reflecting and steering layer for atmospheric gravity waves.

As illustrated by Fig. 3, the distinctive synoptic pattern that occurred in June 2014 affected the entire Mediterranean and Black Sea regions. However, destructive meteotsunamis occurred only in a few specific regions. The governing parameter determining the sea level response to atmospheric disturbances is the *Froude number*, which in the present case can be defined as 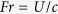; i.e., the ratio of the atmospheric gravity wave speed, *U*, to the phase speed of long ocean waves, 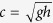, where *g* is the gravity acceleration and *h* is the water depth. Resonance conditions (known as “Proudman resonance”)^21^ occur when 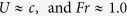. In this case, ocean waves begin to actively absorb atmospheric energy during their propagation and, as a result, are strongly intensified^21^,^22^ (Fig. 4). To determine if such conditions were present during the June 2014 events, we have assumed that 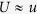 (where *u* is the jet-stream speed at the 500 hPa level estimated from ECMWF operational reanalysis data) and calculated *Fr* for the period 22–27 June but only for areas over which there was an unstable atmospheric layer (*Ri* < 0.25). As indicated by the mapped values of *Fr* in Fig. 1 (the values closest to resonant conditions are plotted), regions with the most favourable conditions for meteotsunami generation (0.9 < *Fr* < 1.1) are in the Adriatic Sea, the Strait of Sicily, the northwestern Black Sea, and a few isolated areas in the Mediterranean. If destructive meteotsunamis are to occur, the width of marine area for which *Fr* ≈ 1.0 has to be sufficiently large in the direction of the atmospheric disturbance propagation to have enough time for the transfer of energy from the atmosphere^22^. For atmospheric disturbances travelling within a northeastward oriented jet stream, as was the case during the June 2014 event, the strongest meteotsunamis are thus expected at the northeastern coasts of resonant areas. It is precisely at these particular locations where the most destructive events occurred. Due to a decrease in water depth and depending on the specific topographical characteristics of the bay or harbour, ocean waves arriving at the coast can continue to amplify and can reach wave heights of several meters^4^,^23^ (Fig. 4).

Although, the June 2014 event is so far the only known Mediterranean-wide destructive meteotsunami, there might have been others. The possibility of the consecutive occurrence of destructive meteotsunamis at distal areas was previously investigated for two of the Mediterranean meteotsunami “hotspots” (the Balearic Islands and the Adriatic Sea)^24^. It was found that high frequency sea level oscillations usually appear at both locations when meteotsunamigenic synoptic patterns propagate from one region to another. However, no case of destructive meteotsunamis occurring at both locations within a span of a few days has yet been found, although more than 20 years of data and eyewitness reports were examined. As evident in Fig. 4, a number of restrictive conditions have to take place to produce a hazardous meteotsunami. As a consequence, even at ”meteotsunami hot spots” these events have a return period of 15 to 20 years^4^,^10^,^25^. It is thus likely that events similar to that of June 2014, with extreme meteotsunamis hitting a number of locations, have even longer return periods. However, this is similar to the case of a *M*_w_ 9.2 earthquake: if it occurred once, then sooner or later it is likely to occur in this region again!

Regardless of the wave formation mechanism, it is clear that there is a real threat from meteotsunamis and that consideration of this threat should be incorporated into tsunami warning procedures and systems. Present day tsunami warning systems do not include monitoring of atmospheric conditions favourable for tsunami generation and are based on: (i) monitoring of seismic activity (tsunami source); and (ii) observation and modelling of the propagation of tsunami waves in the deep ocean and at coastal stations^26^. Addressing the threat from meteorological tsunamis by warning systems requires: (i) monitoring of synoptic conditions (preconditions of the event); (ii) observation, tracking and possible modelling of small-scale air pressure disturbances (monitoring of the meteotsunami source); (iii) observation and modelling of atmosphere-sea interaction (meteotsunami generation, propagation and coastal impact); and (iv) establishment of threshold criteria for warnings, along with protocols and procedures for response. It is noted, however, that due to the stochastic nature of atmospheric gravity waves, this specific warning could be given only after the air pressure disturbance has been observed, i.e. shortly before the wave approaches an endangered area. For this reason, implementation of meteotsunami warning procedures into tsunami warning operations remains a challenge.

## Methods

Frequency-time (*f-t*) diagrams of air pressure and sea level were computed by applying a multi-filter technique, consisting of narrow-band filters and a Gaussian window that isolates a specific centre frequency and demodulates the series to a matrix of amplitudes and phases of wave signals^27^. This method is frequently used for examination of tsunami records and for analyzing tsunami wave energy. All time series, except for the Ninfa air pressure series (6-min time step), were measured with a 1-min sampling. Ninfa air pressure time series were spline interpolated to a 1-min time step for the purposes of clearer presentation. Time series of variance are derived using a 4-hour running average for a 4 day period centred around the time of the event at a given location. The Richardson number, *Ri*, which is a standard measure of atmospheric stability, is given as 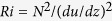 where *N* is the Brunt-Väisälä frequency, *u* is the wind speed and *z* is vertical distance. The *N* frequency was calculated as the moist Brunt-Väisälä frequency^28^ on levels where relative humidity was above 70% or as the dry frequency otherwise. A layer was considered to be dynamically unstable and favourable for trapping of waves in the lower troposphere if *Ri *< 0.25. All input parameters were taken from the European Centre for Middle-range Weather Forecast (ECMWF) operational reanalysis products. Maximum wave heights of sea level oscillations plotted in Fig. 3 are estimated from tide gauge measurements and eyewitness reports for time intervals spanning ±6 hours with respect to the shown reanalysis times.

**Figure 1:**
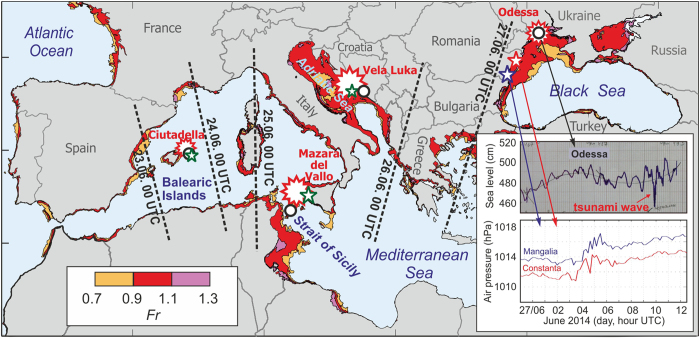
Locations of meteotsunami events observed in the Mediterranean and Black seas during late June 2014 superimposed on meteotsunami-favourable coastal areas for which 0.9 < Fr < 1.1, where Fr is the Froude number. Also shown are the sea level series measured at Odessa and air pressure series measured at two Romanian stations. Small stars mark the positions of air pressure observations; circles denote sea level stations used in Fig. 2. Black dotted lines indicate the approximate onset times of high-frequency sea level oscillations over the Mediterranean and Black seas. The Froude number is defined as the ratio of the wind speed at a height of 500 hPa and the phase speed of long ocean waves. Figure was created using MATLAB software and ECMWF and GEBCO data.

**Figure 3:**
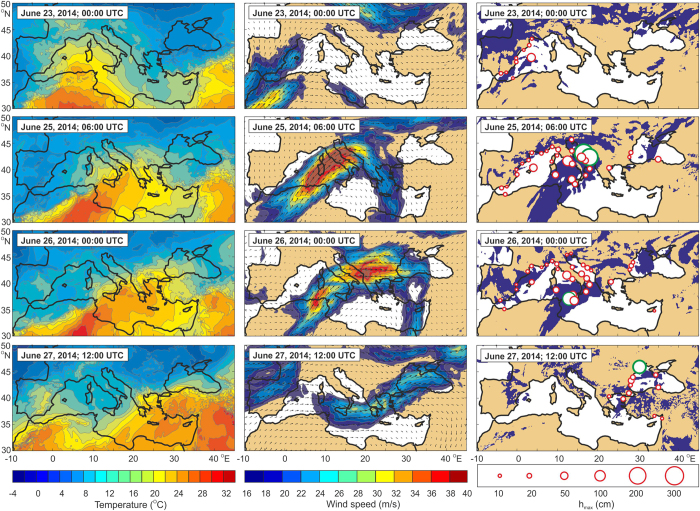
Propagation of the meteotsunamigenic synoptic pattern of June 2014 together with the maximum heights of corresponding sea level oscillations at the times of the meteotsunami events. *Left panel*: temperature at 850 hPa; *middle panel*: wind speed and direction at 500 hPa: and *right panel*: the dynamically instable atmospheric layers (collared denotes *Ri* < 0.25) overlaid by circles showing the maximum height of high-frequency (periods < 3 h) sea level oscillations; the red circles denote measured wave heights, the green circles denote wave heights estimated from videos and eyewitness reports. Figure was created using MATLAB software and ECMWF data.

The Froude number was computed as *Fr* *=* *u/c*, corresponding to the ratio of wind speed *u* (at 500 hPa, the level of ECMWF operational reanalysis data) and the phase speed of long ocean waves, *c*, every 6 hours between 22 and 27 June 2014, and only for those grid points at which the unstable atmospheric layer was present at heights between 700 and 500 hPa. The values closest to resonant conditions (*Fr* ≈ 1.0) are plotted in Fig. 1.

## Additional Information

**How to cite this article**: Šepić, J. *et al.* Widespread tsunami-like waves of 23-27 June in the Mediterranean and Black Seas generated by high-altitude atmospheric forcing. *Sci. Rep.* **5**, 11682; doi: 10.1038/srep11682 (2015).

## Figures and Tables

**Figure 2:**
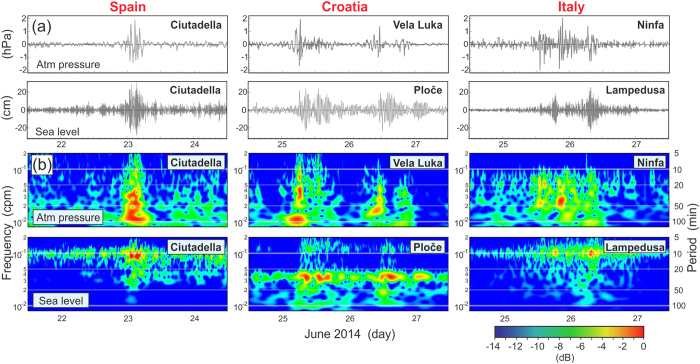
Major atmospheric pressure (AP) and sea level (SL) oscillations measured in various regions of the Mediterranean during 22–27 June 2014. (**a**) AP and SL records after high-pass filtering using a 3-hour Kaiser-Bessel window; and (**b**) frequency-time (*f-t*) diagrams of the records in (**a**).

**Figure 4:**
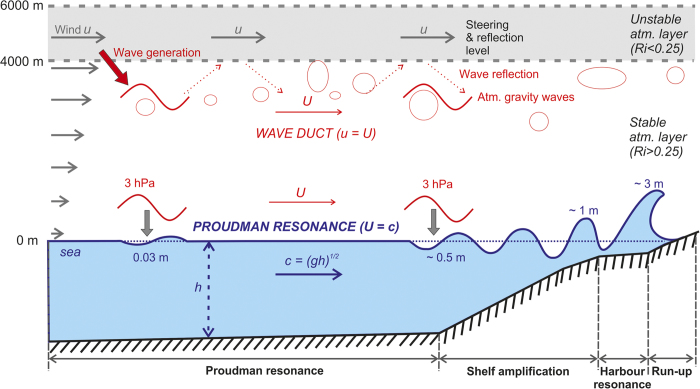
Illustration of the meteotsunami generation processes. Numerous atmospheric gravity waves (represented by bubbles) are generated at the interface of unstable and stable atmospheric layer at places of strong wind shear. Atmospheric gravity wave propagating with speed *U* (represented by the red wave), which is equal to wind speed (*u*) of unstable layer, becomes trapped and propagates in the stable layer as a “duct wave”. Vertical energy loss and dissipation of the duct wave is prevented by an unstable layer. Air pressure change (a surface manifestation of atmospheric gravity waves) generates long-ocean waves which can be amplified through several processes: (1) Proudman resonance (due to matching of long-ocean waves speed and speed of atmospheric gravity wave); (2) shelf amplification (due to shoaling); and (3) harbour resonance (due to matching of frequency of incoming long-ocean waves and harbour eigenperiods). Incoming ocean waves can be amplified more than 100 times before hitting the coast as a destructive meteotsunami. Numbers shown in figure are for illustration only and are highly dependent on the properties of the atmospheric gravity waves, bathymetry and topography of the impacted area.
